# Construction of SNP fingerprint and population genetic analysis of honeysuckle germplasm resources in China

**DOI:** 10.3389/fpls.2023.1080691

**Published:** 2023-03-02

**Authors:** Jianjun Li, Xiaopei Chang, Qian Huang, Pengfei Liu, Xiting Zhao, Fengmei Li, Yungang Wang, Cuifang Chang

**Affiliations:** ^1^ Green Medicine Biotechnology Henan Engineering Laboratory, Engineering Technology Research Center of Nursing and Utilization of Genuine Chinese Crude Drugs in Henan Province, College of Life Science, Henan Normal University, Xinxiang, China; ^2^ School of Life Science and Basic Medicine, Xinxiang University, Xinxiang, China; ^3^ Foresty Seeding Service Station of XinXiang, Xinxiang, Henan, China; ^4^ State Key Laboratory Cell Differentiation and Regulation, College of Life Science, Henan Normal University, Xinxiang, China

**Keywords:** *Lonicera japonica* Thunb., genetic relationship, population structure, SNPs, DNA fingerprint, COVID-19, germplasm resources, SNP markers

## Abstract

**Introduction:**

The flower buds of *Lonicera japonica* Thunb. are widely used in Chinese medicine for their anti-inflammatory properties, and they have played an important role in the fight against SARS COVID-19 and other major epidemics. However, due to the lack of scientific and accurate variety identification methods and national unified standards, scattered and non-standardized management in flower bud production has led to mixed varieties that have caused significant difficulties in the cataloging and preservation of germplasm resources and the identification, promotion, and application of new *L. japonica* varieties.

**Methods:**

In this study, we evaluated the population structure, genetic relationships, and genetic fingerprints of 39 germplasm resources of *Lonicera* in China using simplified genome sequencing technology.

**Results:**

A total of 13,143,268 single nucleotide polymorphisms (SNPs) were identified. Thirty-nine samples of *Lonicera* were divided into four subgroups, and the population structure and genetic relationships among existing *Lonicera* germplasm resources were determined using principal component analysis, population structure analysis, and phylogenetic tree analysis. Through several stringent selection criteria, 15 additional streamlined, high-quality DNA fingerprints were filtered out of the validated 50 SNP loci and verified as being able to effectively identify the 39 *Lonicera* varieties.

**Discussion:**

To our knowledge, this is the first comprehensive study measuring the diversity and population structure of a large collection of *Lonicera* varieties in China. These results have greatly broadened our understanding of the diversity, phylogeny, and population structure of *Lonicera*. The results may enhance the future analysis of genetic diversity, species identification, property rights disputes, and molecular breeding by providing a scientific basis and reference data for these efforts.

## Introduction

*Lonicera japonica* Thunb. a perennial semi-evergreen twining shrub of the Caprifoliaceae family, is native to China and is primarily distributed in temperate regions of the northern hemisphere, with smaller numbers in Japan and Korea. The honeysuckle flower is a dry flower bud or open flower of *L. japonica* and is rich in organic acids, flavonoids (Li et al., 2021), volatile oils, and other components. It can reduce fever and remove toxins without causing gastrointestinal issues (Li, 2020). Especially in China, honeysuckle flower has played an important role in the treatment of SARS COVID-19 and other major epidemics (Ren et al., 2022).

Before 2014, the National Pharmacopoeia Commission of China regarded Flos Lonicerae as a classification under *L. japonica* (Li et al., 2020). The 2015 edition of the Chinese Pharmacopoeia detailed the differences between *L. japonica* and Flos Lonicerae regarding medicinal history, sources, characteristics, chemical components, and other aspects. The medicinal source of honeysuckle flower is *L. japonica*, and the medicinal sources of Flos Lonicerae include *Lonicera macranthoides* Hand.-Mazz., *Lonicera hypoglauca* Miq., *Lonicera confusa* DC., and *Lonicera fulvotomentosa* Hsu et S.C.Cheng. (Zhu et al., 2021). However, it is worth noting that compared with the three Flos Lonicerae, *L. hypoglauca*, *L. confusa*, and *L. fulvotomentosa*, *L. macranthoides* currently has more mature cultivation varieties with the largest number of plantings and the widest planting area, which is more recognized and accepted by the public (Zhang et al., 2022). In the research on Flos Lonicerae, people usually choose *L. macranthoides* as the research object rather than *L. hypoglauca*, *L. confuse*, and *L. fulvotomentosa* (Yao et al., 2022).

With increasing market recognition of L. japonica and L. macranthoides germplasm and the continuous optimization and improvement of breeding technology, the number of Lonicera varieties is increasing (Hu et al., 2022). A total of 39 varieties of *L. japonica* and *L. macranthoides* were authorized or registered in China in April 2022 (Lan, 2017). Most of these varieties are propagated with cuttings and have strong adaptability with no strict soil or climate needs. Sandy loam with a thick soil layer is the best substrate, as it provides an extremely cold-resistant medium (Li et al., 2022). However, due to the lack of standardized management regulations and decentralized management, the *Lonicera* germplasm resources are poorly organized, and there are unclear genetic relationships among varieties. These problems not only lead to intellectual property disputes between varieties but also make it very difficult to catalog and preserve the germplasm resources of *Lonicera* and cultivate new varieties for a wide range of applications. Therefore, accurate and efficient variety identification technology is needed for breeding new high-quality *Lonicera* varieties, and this makes variety identification increasingly important in the breeding industry. At present, the identification of *Lonicera* germplasm is limited to morphological and chemical fingerprint analyses. For example, methods such as high-performance liquid chromatography (HPLC) (Liu et al., 2019) and Fourier transform infrared spectroscopy (FTIR) (Nikzad-Langerodi et al., 2017) have been used to analyze *Lonicera* index components in most studies.

DNA molecular marker technology is widely used in the identification of plant varieties due to its advantages of being free from environmental and species restrictions, its simple operation, and its ability to identify uniform and abundant loci as well as strong polymorphism. In plant biology, simple sequence repeat (SSR) and single nucleotide polymorphism (SNP) markers have been used to construct fingerprint databases, identify genetic relationships, construct genetic maps, and isolate and clone diverse genes of *Nicotiana tabacum* L. (Wang et al., 2021a), *Brassica oleracea* var. *italica* (Shen et al., 2021), *Lagenaria siceraria* (Mol.) Standl. (Wang et al., 2021b), *Triticum aestivum* L. (Li et al., 2018), *Camellia sinensis* L. (Karunarathna et al., 2021), *Beta vulgaris* L. (Schneider et al., 2007), and *Ipomoea batatas* (L.) Lam. (Wang et al., 2010), and other species. Shao et al. (2020) performed genetic detection on 113 olive germplasm resources using eight pairs of SSR fluorescence markers. Zhang et al. (2021) conducted molecular identification and DNA barcode construction of Dracaena germplasm resources from Liliaceae. (Wang et al., 2021a, b) constructed core SNPs to identify calabash germplasm resources using SNP markers, thereby providing data support for the molecular marker-assisted breeding of calabash varieties. SSR and SNP molecular markers have the advantages of rich marker polymorphism, good experimental repeatability, easy standardization of data, clear distribution of marker sites, and mature technology; they are the only recommended markers used to construct a DNA fingerprint database in the Biological and Molecular Marker Technology (BMT) Molecular Test Guidelines of the International Union for the Protection of New Plant Varieties (UPOV) and the General Principles of DNA Fingerprinting Methods for Plant Variety Identification in China (NY/T 2594-2016) (Hayward et al., 2015; Li et al., 2020).

Compared with SSR markers, SNP markers are abundant in number and display greater polymorphism; they are easily detected and convenient for statistical analysis, and they can be identified *via* high-throughput automated detection (Pei et al., 2015). With the continuous improvement of next-generation sequencing (NGS) technology, the development and detection of SNP sites have become simple and efficient. However, in the field of *Lonicera* variety identification, only Peng et al. (2010) designed a pair of allele-specific primers for PCR analysis of nrDNA ITS sequences and successfully distinguished the authenticity of five *L. japonica* species. Xu et al. (2015) used SSR technology to construct SSR fingerprints in Excel format for six different types of *L. japonica*, providing the earliest data support for variety identification. In addition, there have been no studies on the identification of *Lonicera* varieties based on DNA molecular markers (Jiang et al., 2013). The SNP molecular marker technology used in this study refers to the DNA sequence polymorphism caused by the variation of a single base in the genomic DNA sequence (Gazendam et al., 2022). In recent years, an increasing number of studies have used SNP molecular marker technology to identify varieties. DNA fingerprinting technology, with the advantages of being fast and accurate, is a powerful tool for identifying varieties and strains and has been widely used in the diversity and purity identification of many crops (Freixas-Coutin et al., 2019). Therefore, SNP molecular markers are regarded as the most important and promising DNA molecular markers in plant biological research (Azizi et al., 2021).

In this study, 39 *Lonicera* germplasm resources were used for Illumina NovaSeq sequencing. The results were compared with the reference genome to screen and mine SNP core markers and interpret their genetic relationships, genetic diversity, and population structure. In addition, a DNA fingerprint of *Lonicera* was constructed to efficiently and cheaply distinguish different species and varieties of *Lonicera.* The results provide a scientific basis and a data reference for genetic diversity analysis, variety identification, and molecular breeding of *Lonicera* (**Supplementary Figure 1**).

## Materials and methods

### Plant materials

**Figure 1:**
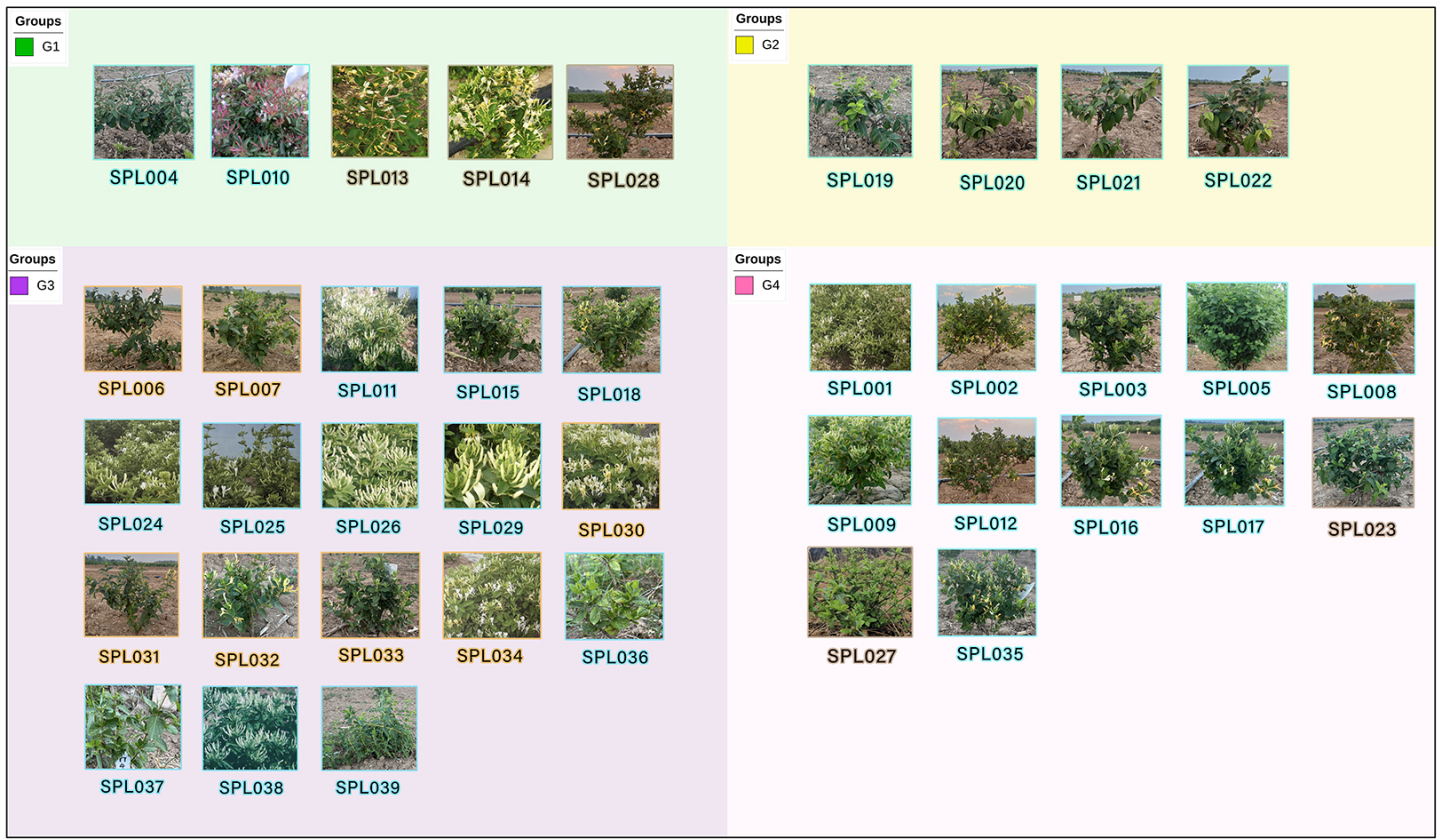
Simplified genome sequencing of 39 wild and cultivated varieties of *L. japonica* and *L. macranthoides* in China. Groups G1, G3, and G4 are *L. japonica* and Group G2 is *L. macranthoides*. Blue represents cultivar, orange represents wild, and brown represents crossbreed.

Considering that *L. macranthoides* in Flos Lonicerae has many cultivated varieties, a wide planting area, and more stable and representative genetic characters. In contrast, *L. hypoglauca*, *L. confuse*, and *L. fulvotomentosa* currently have no cultivated varieties, and there are few wild varieties with low representativeness and other factors. In this experiment, *L. macranthoides* was selected as the representative of Flos Lonicerae. The experiment was conducted with 39 wild and cultivated varieties of *L. japonica* and *L. macranthoides* existing in China. There were 35 varieties of *L. japonica* and four varieties of *L. macranthoides* (**Figure 1**). The specific variety information is shown in **Table 1**. For each germplasm accession, seven vigorous plants were randomly selected at the seedling stage, and young leaf samples totaling 5 g were collected on tinfoil, frozen immediately in liquid nitrogen, and transported to the laboratory, where they were stored at −80°C until they were used for DNA extraction.

**Table 1:** Main information on 39 samples.

Grouping	Number	Variety	Species and genus	Place of origin	Variety type
G1	SPL004	Yate 1	*Lonicera japonica* Thunb.	Shandong	Cultivar (*Lonicera acuminata* Wall.)
G1	SPL010	Yujin 2	*Lonicera japonica* Thunb.	Henan	Cultivar (*Lonicera acuminata* Wall.)
G1	SPL013	Yujin 4	*Lonicera japonica* Thunb.	Henan	Crossbreed (Yujin 2♀× Fengjin 1♂)
G1	SPL014	Yujin 5	*Lonicera japonica* Thunb.	Henan	Crossbreed (Fengjin 1♀×Yujin 2♂)
G1	SPL028	Yujin 5 1-2	*Lonicera japonica* Thunb.	Henan	Crossbreed (Fengjin 1♀×Yujin 2♂)
G2	SPL019	Jincuilei	*Lonicera macranthoides* Hand.-Mazz.	Hunan	Cultivar
G2	SPL020	Yincuilei	*Lonicera macranthoides* Hand.-Mazz.	Hunan	Cultivar
G2	SPL021	Baiyun	*Lonicera macranthoides* Hand.-Mazz.	Hunan	Cultivar
G2	SPL022	Longhua	*Lonicera macranthoides* Hand.-Mazz.	Hunan	Cultivar
G3	SPL006	Mixian xianhua	*Lonicera japonica* Thunb.	Henan	Wild
G3	SPL007	Mixian wild	*Lonicera japonica* Thunb.	Henan	Wild
G3	SPL011	Telei 1	*Lonicera japonica* Thunb.	Henan	Cultivar
G3	SPL015	Mihua 3	*Lonicera japonica* Thunb.	Henan	Cultivar
G3	SPL018	Yujin 3	*Lonicera japonica* Thunb.	Henan	Cultivar
G3	SPL024	Huajin 2	*Lonicera japonica* Thunb.	Shandong	Cultivar
G3	SPL025	Huajin 3	*Lonicera japonica* Thunb.	Shandong	Cultivar
G3	SPL026	Huajin 6	*Lonicera japonica* Thunb.	Shandong	Cultivar
G3	SPL029	Jiufeng 1	*Lonicera japonica* Thunb.	Shandong	Cultivar (Autotetraploid honeysuckle artificially induced from Damaohua)
G3	SPL030	Wildxianhua	*Lonicera japonica* Thunb.	Henan	Wild
G3	SPL031	Changzhenxianhua	*Lonicera japonica* Thunb.	Henan	Wild
G3	SPL032	Xiaojizhua	*Lonicera japonica* Thunb.	Shandong	Wild
G3	SPL033	Dajizhua	*Lonicera japonica* Thunb.	Shandong	Wild
G3	SPL034	Xizhenguanhua	*Lonicera japonica* Thunb.	Shandong	Wild
G3	SPL036	Yate 5	*Lonicera japonica* Thunb.	Shandong	Cultivar
G3	SPL037	Yate 4	*Lonicera japonica* Thunb.	Shandong	Cultivar
G3	SPL038	Fenglei	*Lonicera japonica* Thunb.	Hunan	Cultivar
G3	SPL039	Light red honeysuckle	*Lonicera japonica* Thunb.	Henan	Cultivar (*Lonicera acuminata* Wall.)
G4	SPL001	Fenghua 1	*Lonicera japonica* Thunb.	Henan	Cultivar
G4	SPL002	LuFengwang	*Lonicera japonica* Thunb.	Henan	Cultivar
G4	SPL003	Juhua 1	*Lonicera japonica* Thunb.	Henan	Cultivar
G4	SPL005	Yateliben honeysuckle	*Lonicera japonica* Thunb.	Shandong	Cultivar
G4	SPL008	Mixian Damaohua	*Lonicera japonica* Thunb.	Henan	Wild
G4	SPL009	Yujin 1	*Lonicera japonica* Thunb.	Henan	Cultivar
G4	SPL012	Fengjin 1	*Lonicera japonica* Thunb.	Henan	Cultivar
G4	SPL016	Mihua 2	*Lonicera japonica* Thunb.	Henan	Cultivar
G4	SPL017	Mihua 1	*Lonicera japonica* Thunb.	Henan	Cultivar
G4	SPL023	Longyao	*Lonicera japonica* Thunb.	Hunan	Crossbreed (wild *L. macranthoides*♀×Jincuileei♂)
G4	SPL027	Yujin 6	*Lonicera japonica* Thunb.	Henan	Crossbreed (Telei 1♀×Yujin 1♂)
G4	SPL035	Yateliangzhong	*Lonicera japonica* Thunb.	Shandong	Cultivar
Validation test sample	SPL040	Yateliben	*Lonicera japonica* Thunb.	Shandong	Cultivar
Validation test sample	SPL041	Bainongz	*Lonicera japonica* Thunb.	Henan	Cultivar
Validation test sample	SPL042	Weizi	*Lonicera japonica* Thunb.	Anhui	Cultivar

### DNA extraction and library construction

After grinding the leaf samples, genomic DNA was extracted using a Plant Genomic DNA Kit (TIANGEN, China), and the quality and concentration of DNA were measured using a NanoDrop2000 UV spectrophotometer (Thermo Fisher, Waltham, MA, USA). Then, a paired-end library with a length range of 300–500 bp was constructed *via* double-digested (ddRAD) library construction of qualified sample DNA. First, 500 ng of genomic DNA was reacted with 0.6U EcoRI (NEB), T4 DNA ligase (NEB), ATP (NEB), and EcoRI connectors (including index sequences of differentiated samples) at 37°C for 3 h and annealed at 65°C for 1 h. The restriction enzyme NlaIII (NEB) and the NlaIII connector were then added and allowed to react for 3 h at 37 °C. After the reaction, the endonuclease was inactivated at 65°C for 30 min in a polymerase chain reaction (PCR) amplifier. Digested products of 400–600 bp were recovered *via* agarose gel electrophoresis and quantified using Qubit 3.0 (Life Technology). After mixing 39 samples in equal quantities, an Illumina TruSeq kit was used to construct a DNA library of the mixed products (Hanania et al., 2004).

### Simplified genome sequencing and reference genome alignment

An Illumina NovaSeq 6000 PE150 was used for sample sequencing after library construction, and data quality control was performed on the original sequenced reads (Dellaporta et al., 2016). Fastp software (version: 0.20.0) was used to remove reads with an unknown base number N <5, reads with a length of bases <50%, quality value <5, connector sequences, and other low-quality sequences to obtain clean data. The detailed parameters were set to -q5 -n5. Burrows–Wheeler Aligner (BWA, 0.7.17-R1188) was then used to compare the sequenced reads with the reference genome (Byers et al., 2012). The reference genome used was the GWHAAZE000,000,000 genome Fasta (*L. Lonicera*) (download website: https://ngdc.cncb.ac.cn/search/?dbId=gwh&q=SAMC097356), and the parameters were set as -M -R. The insert size and coverage depth of each sample were counted, and variation was detected by comparing the positions of clean reads on the reference genome (Chao et al., 2009). Then, the same files generated by the comparison were converted to bam format using Samtools software (version: 1.9). Finally, Picard MarkDuplicates (version 2.21.2) was used to detect duplicate tags, and high-quality reads were retained for subsequent analysis.

### SNP analysis

The Genome Analysis Toolkit software (GATK) (https://gatk.broadinstitute.org/hc/en-us; version 4.1.4.1) HaplotypeCaller function was used to call variable outliers. The FilterVCF function was used to filter low-quality mutation sites to obtain the final SNP site set and obtain SNP statistics (Huang et al., 2013). The genome file and the structure annotation gff file were used to annotate the variable outliers using the eff mode in the snpEff software (version 4.3t) (Pariasca-Tanaka et al., 2015). SNPs and INDELs were screened according to the following criteria (Elbasyoni et al., 2018): average sequencing depth ≥5× (Davey et al., 2011), minor allele frequency (MAF) ≥0.05, information integrity ≥0.70, SNP quality value Q ≥30, QD <2.0, MQ <40.0, FS >60.0, SOR >6.0, MQRankSum <−12.5, and ReadPosRankSum <−8.0 (Angel et al., 2012).

### Genetic diversity and population structure analysis

Based on the high-quality SNPs obtained, GCTA software (version 1.92.1, http://cnsgenomics.com/software/gcta/#Overview) was used to perform principal component analysis (PCA) (Abdelhafez et al., 2019) with the following parameter settings: – make grm – autosome (Gong et al., 2016). The maximum likelihood (ML) method in FastTree software (version 2.1.9) was used to construct an evolutionary tree of 39 *Lonicera* samples (Saxena et al., 2012). Finally, admixture software (Sorkheh et al., 2007) (version 1.3.0) was used to analyze the population genetic structure (Jaganathan et al., 2015).

### Fingerprint construction

DNA fingerprint construction was performed based on the high-quality SNPs obtained (Xu et al., 2017). As few markers as possible were used to identify as many varieties as possible to achieve the purposes of simplicity, efficiency, and economy (Wang et al., 2009). Fifty core markers with a high detection rate and significant amounts of polymorphism that could distinguish all varieties were screened out based on the size of the polymorphic information content (PIC) value and distribution frequency (Pavana et al., 2012), and then a DNA fingerprint was constructed. Furthermore, the 15 leanest SNP combinations were further screened out (Jones and Mackay, 2015) to identify *Lonicera* varieties with lower costs and faster speeds.

### Verification of SNP locus authenticity

Primers for the 15 SNP loci were designed using Primer Premier 5 software (**Table 2**), and the DNA of the validation test sample was extracted to conduct PCR. The parameters were set as follows: length, 18–30 bp; Tm value, 55–65 degrees; and GC content, 40%–70%. The primers were synthesized by Sangon Bioengineering Co., Ltd. (Shanghai, China). Four samples were randomly selected from 39 known samples, and three unknown samples were added (**Table 1**). A total of seven verification DNA samples were selected as templates for PCR amplification. The total volume of the PCR mixture was 25 μl, containing 1 μl DNA template (100 ng/μl), 1 μl each of Primer F and Primer R (10 pmol/L), 0.2 μl Taq Plus DNA polymerase (5 U/μl), 2.5 μl 10× PCR buffer with Mg^2+^, and 1 μl dNTP (10 mM). The PCR reaction program involved pre-denaturation at 95°C for 5 min, denaturation at 94°C for 30 s, annealing for 30 s to 63°C, and extension at 72°C for a total of 30 s; this was repeated for 30 cycles, after which there was a final extension at 72°C for 10 min before storage at 4°C. After PCR amplification, 5 μl of PCR products were taken and subjected to 1% agarose gel electrophoresis at 150 V and 100 mA. After 10–20 min of observation, the target PCR band was cut from the gel, recovered, and then sequenced using 3730XL. The sequencing data were searched in the results group, and sequence analysis software SnapGene was used for analysis. Finally, SeqMan software was used to observe the peak map to test the reproducibility, genetic stability, and specificity of 15 SNPs.

**Table 2:** Primer information.

Number	Primer sequences	Product length/bp
Marker1	F:TTGAGATGAACCGAGTTAGGG R:GCAGCCTGACCAAACAGTTC	258
Marker2	F:ACGGGCACATCAGGAGAC R:AGAATATTTTGATAATCCACACG	295
Marker3	F:TCATTCCAGGGATCTAAATTGG R:GGTGGGATTTGTTAATCATCG	284
Marker4	F:GCATCAAGGTGTTCATAGAACTG R:CTTCGACACAATCCATGTCAC	280
Marker5	F:TTGGGAGAGGAGGATTTGAG R:TCCCAGCTCTTACGTTGGTC	295
Marker6	F:GGCAAGAGATTTGGTCAAAGG R:GAATTCCATGCCTAGTGTTCG	273
Marker7	F:TAAGAGGAAAACTATGAACATGTCG R:ATAACATTTAGAATTGCCTACTCCC	267
Marker8	F:AGAGACTACTCAAATAAATGTGGGC R:CTTTACAAGGCGATTATAGTTTTTG	238
Marker9	F:CTTCTTGGGATGTGTGTAGGG R:AAGAAGTGTTCCTGCACCTTG	293
Marker10	F:TTTTATTCACCCAATAATAAGCGAG R:AGTCCATCAAAGTAGCTTGCTATTG	199
Marker11	F:GCAAGATCCCACACTTCTGTC R:CATTTGCACCAGCCATTC	291
Marker12	F:CCTGCTTACCAACACCTTGC R:TGAGGTTTCCACCTTCCATC	286
Marker13	F:GGACTGCTTGCTGAATCTCC R:GTGCAAACAAGGGCCAAG	289
Marker14	F:TTCAATCATCTCCGACAAGAAG R:AAGTGGTATGTGTTGCCTTTAG	275
Marker15	F:TTCTTGGAATGGCTGTTGTG R:AGAAAACGGAATTGCTCCAG	290

## Results

### Simplified genome sequencing and reference genome alignment

The sequencing of 39 *Lonicera* samples on the Illumina NovaSeq 6000 PE150 yielded a total of 84.88 Gb of clean data, and Q30 reached more than 93.25% per sample (**Table 3**). The obtained clean reads were mapped to the reference genome, and the mapping efficiency reached 99.38% (**Table 4**).

**Table 3:** Evaluation of sample sequencing data.

Sample ID	Read Number	Base Number	Q30 (%)	Q20 (%)	Average Q
SPL001	7,185,184	2,069,332,992	91.28	96.92	35.52
SPL002	6,769,839	1,949,713,632	93.07	97.63	35.84
SPL003	6,278,751	1,808,280,288	93.07	97.63	35.84
SPL004	6,501,683	1,872,484,704	93.23	97.7	35.87
SPL005	6,239,695	1,797,032,160	92.81	97.53	35.79
SPL006	6,373,808	1,835,656,704	93.11	97.65	35.84
SPL007	6,160,043	1,774,092,384	93.1	97.64	35.84
SPL008	8,059,394	2,321,105,472	93.12	97.65	35.85
SPL009	5,975,506	1,720,945,728	93.05	97.61	35.83
SPL010	6,844,368	1,971,177,984	93.14	97.66	35.85
SPL011	5,876,620	1,692,466,560	92.75	97.52	35.78
SPL012	8,432,240	2,428,485,120	93.15	97.67	35.85
SPL013	7,983,793	2,299,332,384	93.21	97.7	35.86
SPL014	8,386,930	2,415,435,840	93.02	97.6	35.83
SPL015	7,378,404	2,124,980,352	92.95	97.57	35.81
SPL016	6,530,387	1,880,751,456	93.14	97.67	35.85
SPL017	7,223,326	2,080,317,888	93.01	97.6	35.82
SPL018	7,222,587	2,080,105,056	92.75	97.53	35.78
SPL019	6,961,363	2,004,872,544	92.96	97.57	35.81
SPL020	6,239,014	1,796,836,032	93.25	97.72	35.87
SPL021	8,513,100	2,451,772,800	91.35	96.95	35.54
SPL022	7,453,392	2,146,576,896	93.13	97.65	35.85
SPL023	7,979,692	2,298,151,296	93.06	97.62	35.83
SPL024	8,625,188	2,484,054,144	93.24	97.71	35.87
SPL025	7,576,097	2,181,915,936	92.88	97.55	35.8
SPL026	8,546,185	2,461,301,280	93.1	97.64	35.84
SPL027	9,432,650	2,716,603,200	93.05	97.62	35.83
SPL028	8,520,406	2,453,876,928	93.18	97.67	35.86
SPL029	8,074,251	2,325,384,288	93.12	97.65	35.85
SPL030	6,953,860	2,002,711,680	92.63	97.47	35.76
SPL031	9,462,603	2,725,229,664	93.22	97.7	35.86
SPL032	9,550,614	2,750,576,832	93.17	97.68	35.86
SPL033	6,736,938	1,940,238,144	93.18	97.67	35.86
SPL034	3,089,989	889,916,832	92.91	97.55	35.81
SPL035	9,336,356	2,688,870,528	92.98	97.58	35.82
SPL036	9,054,360	2,607,655,680	93.18	97.69	35.86
SPL037	8,583,574	2,472,069,312	93.07	97.62	35.84
SPL038	8,127,226	2,340,641,088	92.7	97.51	35.78
SPL039	10,499,418	3,023,832,384	93.23	97.71	35.87

Read Number, the total number of paired-end reads in the clean data; Base Number, clean data; Q30 (%), the percentage of bases whose clean data quality value is greater than or equal to 30; Q20 (%), the percentage of bases whose clean data quality value is greater than or equal to 20; Average Q, average quality value.

**Table 4:** Statistics of sample map results.

Sample ID	Total Reads	Genome Size	Cover Size	Cover Bases	Mapped Reads
SPL001	14,370,368	903,813,177	103,257,927	2,050,379,568	14,238,747 (99.08%)
SPL002	13,539,678	903,813,177	85,860,469	1,947,053,376	13,521,204 (99.86%)
SPL003	12,557,502	903,813,177	128,173,474	1,804,225,104	12,529,341 (99.78%)
SPL004	13,003,366	903,813,177	98,669,372	1,865,118,816	12,952,214 (99.61%)
SPL005	12,479,390	903,813,177	91,976,345	1,790,563,392	12,434,468 (99.64%)
SPL006	12,747,616	903,813,177	104,056,906	1,820,330,640	12,641,185 (99.17%)
SPL007	12,320,086	903,813,177	103,665,687	1,747,244,592	12,133,643 (98.49%)
SPL008	16,118,788	903,813,177	82,455,079	2,304,494,352	16,003,433 (99.28%)
SPL009	11,951,012	903,813,177	87,040,006	1,709,987,616	11,874,914 (99.36%)
SPL010	13,688,736	903,813,177	97,046,865	1,961,574,480	13,622,045 (99.51%)
SPL011	11,753,240	903,813177	76,259,815	1,688,009,040	11,722,285 (99.74%)
SPL012	16,864,480	903,813,177	113,488,986	2,424,669,552	16,837,983 (99.84%)
SPL013	15,967,586	903,813,177	112,032,425	2,290,615,344	15,907,051 (99.62%)
SPL014	16,773,860	903,813,177	112,673,425	2,403,909,936	16,693,819 (99.52%)
SPL015	14,756,808	903,813,177	104,751,936	2,109,854,736	14,651,769 (99.29%)
SPL016	13,060,774	903,813,177	89,005,257	1,862,137,296	12,931,509 (99.01%)
SPL017	14,446,652	903,813,177	91,238,406	2,071,778,544	14,387,351 (99.59%)
SPL018	14,445,174	903,813,177	93,874,630	2,076,098,400	14,417,350 (99.81%)
SPL019	13,922,726	903,813,177	83,487,576	1,983,965,904	13,777,541 (98.96%)
SPL020	12,478,028	903,813,177	68,650,762	1,777,603,824	12,344,471 (98.93%)
SPL021	17,026,200	903,813,177	129,987,788	2,418,935,184	16,798,161 (98.66%)
SPL022	14,906,784	903,813,177	93,547,130	2,112,933,888	14,673,152 (98.43%)
SPL023	15,959,384	903,813,177	160,674,767	2,286,710,064	15,879,931 (99.50%)
SPL024	17,250,376	903,813,177	97915450	2,477,152,224	17,202,446 (99.72%)
SPL025	15,152,194	903,813,177	75921750	2,174,709,600	15,102,150 (99.67%)
SPL026	17,092,370	903,813,177	119,762,358	2,449,613,376	17,011,204 (99.53%)
SPL027	18,865,300	903,813,177	122,060,515	2,711,293,776	18,828,429 (99.80%)
SPL028	17,040,812	903,813,177	108,119,689	2,442,139,056	16,959,299 (99.52%)
SPL029	16,148,502	903,813,177	100,179,219	2,320,033,392	16,111,343 (99.77%)
SPL030	13,907,720	903,813,177	91,660,453	1,996,836,048	13,866,917 (99.71%)
SPL031	18,925,206	903,813,177	111,541,183	2,716,367,616	18,863,664 (99.67%)
SPL032	19,101,228	903,813,177	125,378,619	2,724,372,720	18,919,255 (99.05%)
SPL033	13,473,876	903,813,177	102,814,097	1,922,729,328	13,352,287 (99.10%)
SPL034	6,179,978	903,813,177	53,108,193	887,653,008	6,164,257 (99.75%)
SPL035	18,672,712	903,813,177	118,934,312	2,684,102,544	18,639,601 (99.82%)
SPL036	18,108,720	903,813,177	106,810,410	2,588,668,560	17,976,865 (99.27%)
SPL037	17,167,148	903,813,177	91,477,786	2,433,739,104	16,900,966 (98.45%)
SPL038	16,254,452	903,813,177	103,563,755	2,316,495,456	16,086,774 (98.97%)
SPL039	20,998,836	903,813,177	132,956,199	3,006,005,472	20,875,038 (99.41%)

Total Reads refers to the total number of reads for sequencing, and a pair of paired-end reads refers to two counts of reads; Genome Size is the genome size; Cover Size is the size of covering genome; Cover Bases is the base number of the sequencing coverage; Mapped Reads is the number of reads in the map.

### Selection and identification of high-quality SNPs

**Figure 2:**
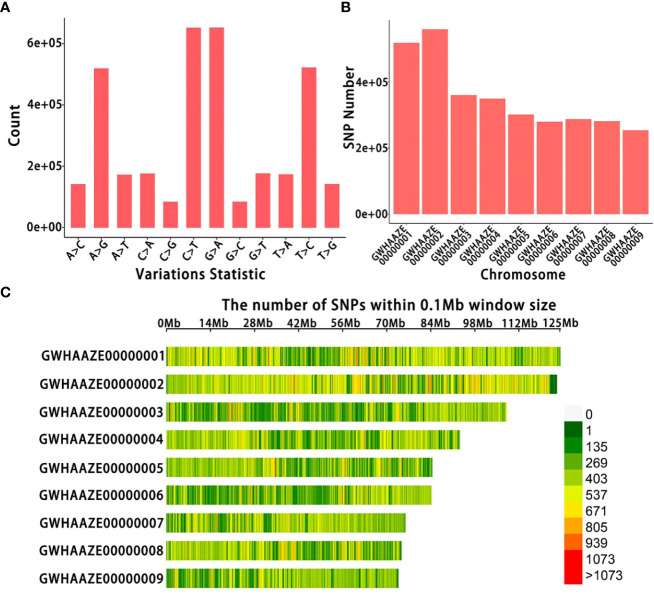
Single nucleotide polymorphism (SNP) identification of 39 *Lonicera* samples. **(A)** Number of SNP types. The horizontal axis represents the different types of SNP mutations, and the vertical axis represents the number of mutations. **(B)** Number of SNPs on each chromosome. The horizontal axis represents the chromosome number, and the vertical axis represents the number of SNPs. **(C)** SNP density distribution on each chromosome. The horizontal axis represents the chromosome length, and the vertical axis represents the chromosome number. Different colors represent the number of SNPs in different regions.

After sequencing, GATK software detected many SNP variants in 39 *Lonicera* samples; sequencing generated a total of 13,143,268 SNPs (**Figure 2A**). A total of 3,374,929 filtered SNPs were obtained, distributed on nine chromosomes (**Figure 2B**). Finally, the SNP distribution map on each chromosome was drawn according to the number and density of SNPs (**Figure 2C**). There were about 400,000 SNPs on chromosome GWHAAZE000000000 1 and 600,000 SNPs on chromosome GWHAAZE000000000 2. For chromosomes GWHAAZE000000000 3 to 9, the numbers of SNPs on these six chromosomes were similar, and there were about 300,000 SNPs on each chromosome.

### Genetic relationships and population structure analysis

**Figure 3:**
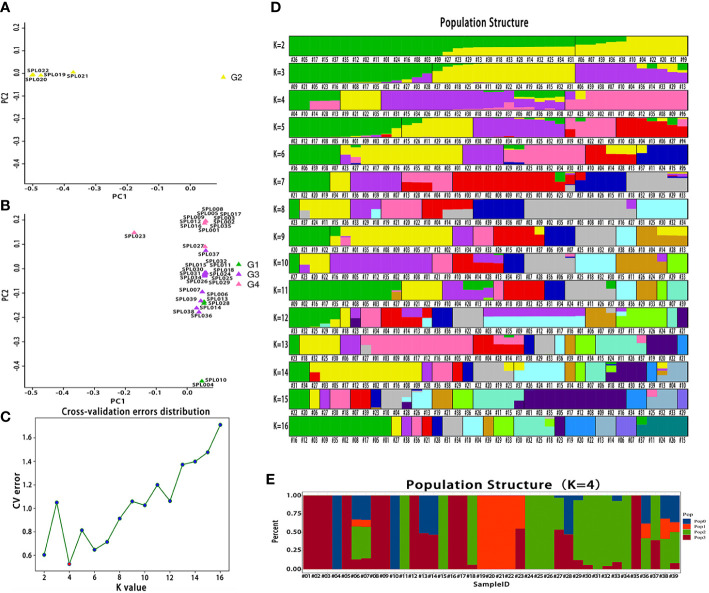
Bioinformatic analysis of 39 *Lonicera* varieties based on single nucleotide polymorphisms (SNPs). **(A)** A two-dimensional diagram of principal component analysis (PCA). **(B)** The cross-validation error rate corresponding to different K values. **(C)** Population structure of 39 *Lonicera* varieties at different K values. The K value represents the cross-validation error rate. **(D)** Population structure of 39 *Lonicera* samples when K = 4.

The PCA of high-quality SNPs screened from 39 *Lonicera* samples was conducted using GCTA software. *L. macranthoides* samples from Hunan were clearly clustered on one side (**Figure 3A** and **Table 1**), while *L. japonica* samples from the same species were clustered on the other side (**Figure 3B** and **Table 1**). For the two clusters classified by species, detailed clustering classification was further conducted according to a variety of characteristics of different *Lonicera* samples. For example, honeysuckle varieties with red flower buds were clustered together. Crossbred samples were completely clustered together. Admixture software was used to analyze the population structure of the 39 *Lonicera* samples. The cross-validation error rate was the lowest when K = 4, a result that was consistent with the variety characteristics and source classification of the 39 *Lonicera* samples (**Figure 3C** and **Table 1**). The 39 *Lonicera* germplasm resources were divided into four subgroups (**Figures 3D, E**).

**Figure 4:**
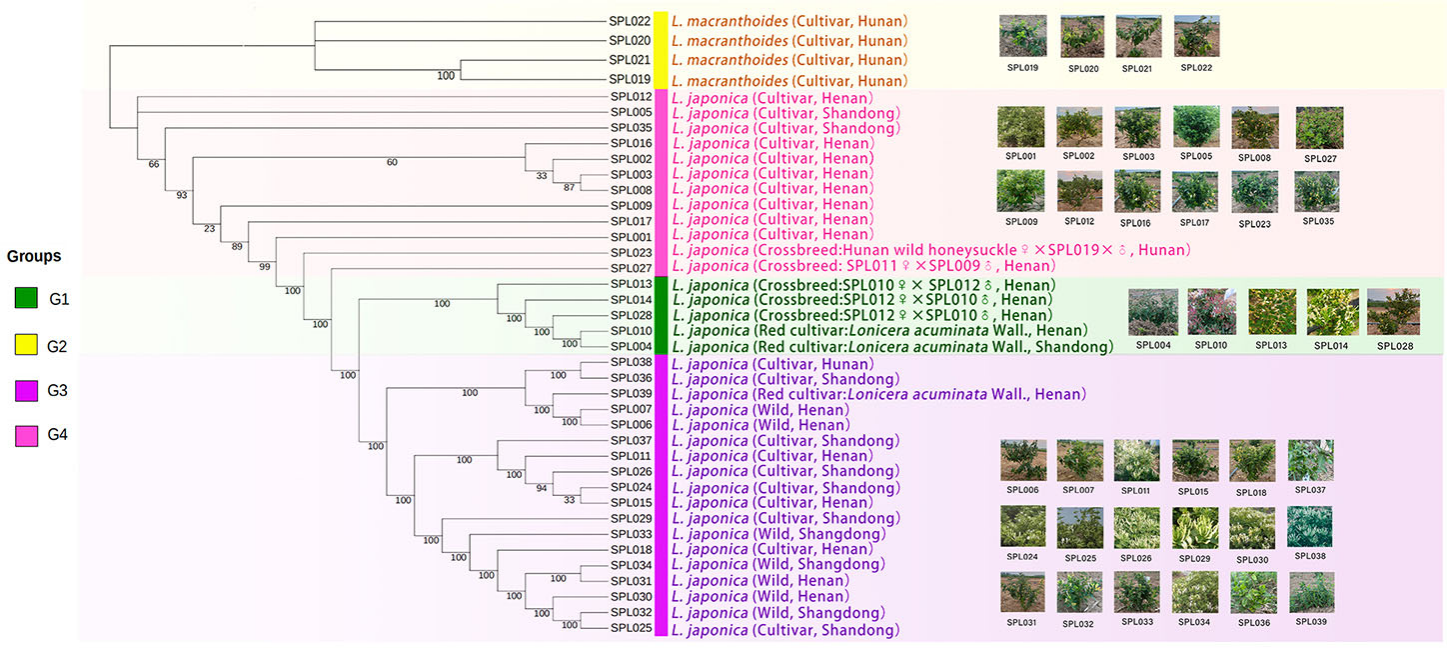
Phylogenetic tree of 39 *Lonicera* varieties. G1 is a *L. japonica* variety with red and light red flower buds; outgroup G2 is *L. macranthoides*; G3 is mainly wild *L. japonica* from Shandong and Henan; and G4 is a mature and highly recognized *L. japonica* in the current market. The text annotation in the figure is: species name (species type, origin).

Finally, FastTree software was used to construct an evolutionary tree, and the 39 *Lonicera* samples were clustered into four groups (**Figure 4**), consistent with the results of the PCA and the population structure analysis. In addition, through further analysis, we found that the four clusters also had high similarity in phenotype, species, and origin. Each group was supported by a high bootstrap value.

### Construction of a DNA fingerprint

**Figure 5:**
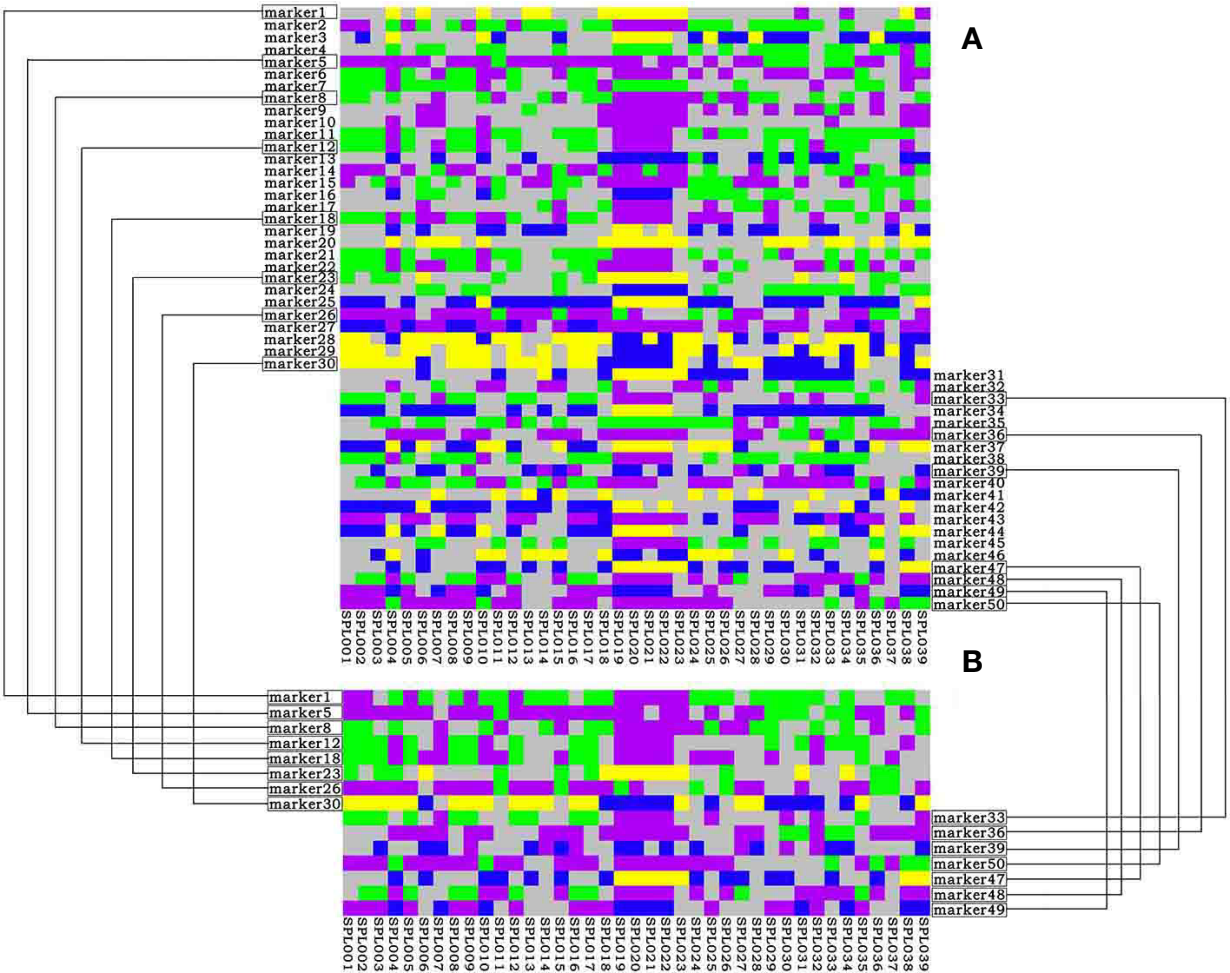
DNA fingerprinting of 39 *Lonicera* samples. **(A)** DNA fingerprint is composed of 50 single nucleotide polymorphisms (SNPs). **(B)** DNA fingerprint composed of 15 SNPs. Homozygous genotypes C/C, A/A, T/T, and G/G are represented by yellow, green, blue, and purple, respectively; heterozygous genotypes are represented by gray; deletion genotypes are represented by white.

Based on the results of simplified sequencing, SNP loci with a PIC value greater than 0.30 that were uniformly distributed on nine chromosomes were screened as the core loci for the construction of the *Lonicera* variety fingerprint. Finally, 50 SNP loci were selected for constructing the fingerprint, and all varieties were distinguished (**Figure 5A**). Among the 50 core SNPs, loci Markers 8, 17, 23, and 32 had the highest PIC value of 0.38. Marker 4 had the lowest PIC value of 0.30. The average PIC value was 0.35, indicating moderate polymorphism. Using this set of core SNP loci combinations, 39 samples of *Lonicera* were compared in pairs. The statistical results for the number of different loci between the samples showed many different loci between each pair. To quickly and cheaply distinguish *Lonicera* varieties, in this study we screened out the combination with the minimum number of SNPs that could distinguish 39 *Lonicera* varieties from the obtained 50 core loci; we obtained the most simplified SNP combination that contained 15 SNPs distributed on eight chromosomes (**Figure 5B**). When using the combination of 15 SNPs to distinguish samples, there was at least one different SNP between each of the two samples that could effectively distinguish the 39 *Lonicera* varieties.

Some *Lonicera* samples could only be distinguished based on one SNP. For example, “Longhua” (SPL022), “Yincuilei” (SPL019), and “Jincuilei” (SPL020) could only be distinguished by Marker 7; “Yujin 2” (SPL010) and “Yate 1” (SPL004) could only be distinguished by Marker 6. “Yujin 5” (SPL014) and “Yujin 5 1-2” (SPL028) could only be distinguished by Marker 11. These results also illustrate that it is difficult to develop fingerprints against the background of complex and chaotic genetic relationships in *L. japonica* germplasm.

### Verification of SNP loci and genetic stability of honeysuckle

We randomly selected four samples, “Yujin 1,” “Yujin 2,” “Mixian Damaohua,” and “Telei 1,” from a set of 39 samples to verify the reproducibility of SNPs. Fifteen SNPs had the same site specificity as the previous test results (**Table 5**), with a clear and clutter-free locus peak map, 100% reproducibility, and ideal results. Next, we tested the genetic stability of 2-, 5-, 7-, 8-, 9-, and 10-year-old plants of “Yujin No. 1.” The results showed that the genetic stability of honeysuckle of the same variety in different years was as high as 97.33% (**Table 6**). Finally, three new varieties, “Yateliben,” “Bainong 2,” and “Weizi,” were added to verify the specificity of 15 SNP loci (**Table 7**). The results showed that 15 SNP loci could effectively distinguish the genotypes of the newly added varieties (**Supplementary Figure 2**).

**Table 5:** Verification results of 15 single nucleotide polymorphism (SNP) sites.

Marker	Yujin 1 (REF/ALT)	Yujin 2 (REF/ALT)	Mixian Damaohua (REF/ALT)	Telei 1 (REF/ALT)
Marker 1	G/G	A/G	A/A	A/A
Marker 2	G/G	G/G	G/G	A/A
Marker 3	R/A	A/A	R/G	G/G
Marker 4	A/A	G/G	A/A	R/G
Marker 5	A/A	G/G	A/A	G/G
Marker 6	M/A	M/A	M/A	A/C
Marker 7	G/G	M/A	G/G	A/A
Marker 8	C/C	G/G	C/C	Y/C
Marker 9	A/A	C/A	A/A	R/G
Marker 10	G/R	G/G	R/G	R/A
Marker 11	G/G	K/G	K/G	K/G
Marker 12	G/G	A/A	G/G	G/G
Marker 13	R/A	G/G	M/C	G/G
Marker 14	Y/C	T/T	Y/C	Y/C
Marker 15	T/T	C/C	T/T	Y/C

Marker is the name of the SNP site; REF is the reference genome genotype; ALT is the verification result; genotype: R = A/G, Y = C/T, M = A/C, K = G/T, S = C/G, W = A/T.

**Table 6:** *L. japonica* genetic stability test.

Number	YJ01 (REF/ALT)	YJ02 (REF/ALT)	YJ03 (REF/ALT)	YJ04 (REF/ALT)	YJ05 (REF/ALT)	YJ06 (REF/ALT)	YJ07 (REF/ALT)	YJ08 (REF/ALT)	YJ09 (REF/ALT)	YJ10 (REF/ALT)
Marker1	A/G	A/G	A/G	A/G	A/G	A/G	A/G	A/G	A/G	A/G
Marker2	G/G	G/G	G/G	G/G	G/G	G/G	G/G	G/G	G/G	G/G
Marker3	A/A	A/A	A/A	A/A	A/A	A/A	A/A	A/A	A/A	A/A
Marker4	A/A	A/A	A/A	A/A	A/A	A/A	A/A	A/A	A/A	A/A
Marker5	A/A	A/A	A/A	A/A	A/A	A/A	A/A	A/A	A/A	A/A
Marker6	T/T	T/T	T/T	T/T	T/T	T/T	T/T	T/T	T/T	T/T
Marker7	G/G	G/G	G/G	G/G	G/G	G/G	G/G	G/G	G/G	G/G
Marker8	C/C	C/C	C/C	C/C	C/C	C/C	C/C	C/C	C/C	C/C
Marker9	A/A	A/A	A/A	A/A	A/A	A/A	A/A	A/A	A/A	A/A
Marker10	A/G	A/G	A/G	A/A	A/A	A/A	A/G	A/A	A/A	A/A
Marker11	T/G	T/G	T/G	T/G	T/G	T/G	T/G	T/G	T/G	T/G
Marker12	G/G	G/G	G/G	G/G	G/G	G/G	G/G	G/G	G/G	G/G
Marker13	T/T	T/T	T/T	T/T	T/T	T/T	T/T	T/T	T/T	T/T
Marker14	A/A	A/A	A/A	A/A	A/A	A/A	A/A	A/A	A/A	A/A
Marker15	G/G	G/G	G/G	G/G	G/G	G/G	G/G	G/G	G/G	G/G

Marker is the name of the SNP site; REF is the reference genome genotype; ALT is the verification result.

**Table 7:** Fifteen single nucleotide polymorphism (SNP) site specificity verification.

Marker	Yateliben (REF/ALT)	Bainong 2 (REF/ALT)	Weizi (REF/ALT)
Marker 1	A/G	A/A	A/G
Marker 2	G/G	G/G	G/G
Marker 3	A/G	G/G	A/A
Marker 4	A/A	A/G	A/G
Marker 5	A/A	G/G	A/G
Marker 6	T/T	T/T	T/T
Marker 7	G/G	A/A	G/G
Marker 8	C/C	T/C	C/C
Marker 9	A/A	A/G	A/A
Marker 10	A/G	A/A	A/A
Marker 11	T/G	T/G	T/T
Marker 12	G/G	G/G	A/A
Marker 13	T/T	T/T	T/T
Marker 14	A/A	G/G	G/G
Marker 15	G/G	T/G	T/G

Marker is the name of the SNP site; REF is the reference genome genotype; ALT is the verification result; genotype: R = A/G, Y = C/T, M = A/C, K = G/T, S = C/G, W = A/T.

## Discussion

### SNP-based genetic relationships among the *Lonicera*

As the original and major producer of *Lonicera*, China has three main production areas: Fengqiu in Henan Province, Julu in Hebei Province, and Pingyi in Shandong Province (Song, 2020). The planting area of *L. japonica* in 2022 reached 1,066.7 km^2^ (Lv, 2020; Ma et al., 2022), with an annual output value of nearly 10 billion CNY (Liu, 2021; Liu, 2022). At present, many *Lonicera* varieties have been widely planted, but there has been a lack of standardized and decentralized management. Therefore, it was necessary to clarify the genetic relationship and population structure of existing *Lonicera* varieties.

The 2015 Edition of the Chinese Pharmacopoeia details the differences between *L. japonica* and Flos Lonicerae in medicinal history, sources, characteristics, chemical components, and other aspects, showing that Flos Lonicerae is classified as *L. macranthoides*, *L. hypoglauca*, *L. confusa*, and *L. fulvotomentosa. L. macranthoides* is more recognized because of its easy cultivation, large number of plants, and wide planting area. Lu et al. (2017) established the fingerprints of five batches of *L. japonica* and three batches of *L. macranthoides* using proton nuclear magnetic resonance (1H-NMR) that provided a basis for the quality control analysis of *L. japonica* and *L. macranthoides*. Ren et al. (2022) used microscopy and thin-layer chromatography (TLC) to evaluate the quality of *L. japonica* varieties in Henan, Hebei, Shandong, and other areas and concluded that the index components of Shandong *L. japonica* were the highest. This work provided a scientific basis for the selection of raw materials for honeysuckle decoctions. Deng et al. (2021) conducted a comparative study on tree-shaped and wild-type *L. japonica* using HPLC fingerprints and found that wild *L. japonica* contained more chemical components, while tree-shaped *L. japonica* was more consistent. Li et al. (2020) conducted a comparative analysis of the characteristics and pharmacological effects of *L. japonica* and *L. macranthoides*, providing data support for their application in traditional Chinese medicine. The related studies focused on *Lonicera* in the early stages; most of the studies on the germplasm focused on morphological analysis, index component differences, and quality evaluation among varieties, while there was almost no research on the genetic relationships and population structure of *Lonicera* species and varieties.

In this study, 39 existing *Lonicera* germplasm resources in China were collected and divided into four subgroups *via* simplified sequencing, PCA, population structure analysis, and evolutionary tree construction. Subgroup I contains the red variety *L. acuminata* of *L. japonica*. Subgroup II contained *L. macranthoides*. Subgroup III comprises the main, wild, and hybrid varieties of *L. japonica* from Shandong and Henan. Subgroup IV contained cultivars of *L. japonica* that comprised varieties with high yields, strong resistance to pests and diseases, and good characteristics that have been recognized on the current market, for example, SPL009, SPL002, SPL003, and other varieties. Based on these results, it should be clear that the lineages of wild *Lonicera* species were extremely complex, while the lineages of systematically bred *L. japonica* were relatively simple. For example, “Mixian xianhua” (SPL006), “Mixian wild” (SPL007), and “Mixian Damaohua” (SPL039) are wild varieties of *L. japonica* with extremely complex genetic relationships. In the population structure analysis, they all had ancestral lines from the four subgroups. However, the genetic relationship between “Yujin 1” (SPL009) and “Yate liangzhong” (SPL035) was simple and derived from one pedigree. After further analysis, this study also clarified the controversial genetic relationship of “Longyao” (SPL023), a variety produced in Hunan, as belonging to *L. macranthoides*. However, our results confirmed that “Longyao” (SPL023) was a cross-species hybrid of a female parent *L. japonica* and a male parent *L. macranthoides*, which explained why it simultaneously had the pedigree of two subgroups in the PCA and population structure analysis. The genetic relationship of “Yujin 6” (SPL027) was further clarified, and the results showed that it was crossbred from the female parent “Telei 1” (SPL011) and the male parent “Yujin 1” (SPL009). This finding well explained its position in Group IV of the evolutionary tree. In addition, four other hybrids were used in this study. Population structure analysis showed that all hybrids have genetic genes from their parents, which was largely consistent with expectations. At present, this research group is conducting a more in-depth analysis of hybrid characteristics.

### Identification of 39 *Lonicera* germplasm resources by SNP fingerprints

Different from most previous studies focusing on honeysuckle morphology and quality evaluation (Li et al., 2022), the present study focused on aspects including analyzing the population structure of *L. japonica*, excavating the differences among varieties of *L. japonica*, and using SNP to distinguish varieties of *L. japonica*. This work can not only explain the population relationships of existing *Lonicera* germplasm resources but can also further clarify the genetic relationships between *Lonicera* varieties. These results provide a favorable reference and a basis for the management of *Lonicera* germplasm resources and have important significance for the breeding of new *Lonicera* varieties in the future.

In this study, SNP molecular marker technology and DNA fingerprinting technology were used; 3,374,929 SNPs were obtained from 39 samples of *Lonicera*; 50 core SNPs with a strong ability to identify *Lonicera* germplasm were screened; and DNA fingerprints were constructed. A fingerprint map constructed with 50 core SNP markers effectively identified and distinguished differences among different species and varieties of *Lonicera*. In addition, this study further filtered out 15 SNPs as the most compact site combination. The DNA fingerprints constructed based on the 15 SNP loci were used to detect different *Lonicera* samples, and ≥1 different locus was found in each *Lonicera* variety that could be used to distinguish different species and varieties rapidly, accurately, and at a low cost.

Considering the extremely complex genetic background of *Lonicera* germplasm, this study conducted further research on varieties that had several different loci ≤1 in the fingerprints of 15 SNP loci. After further analysis, it was found that these varieties not only had high repeatability in DNA fingerprints but also had high similarity in origin, species, and characteristics. For example, “Mihua 1” (SPL017) and “Fengjin 1” (SPL012) were introduced and domesticated from “Mixian Damaohua” (SPL008). The varieties “Longhua” (SPL022), “Jincuilei” (SPL020), and “Yincuilei” (SPL019) originated from Hunan Province and were *L. macranthoides* “Yujin 2” (SPL010) and “Yate 1” (SPL004) had purplish red flower buds and were red varieties of *L. japonica*. “Yujin 5” (SPL014) and “Yujin 5 1-2” (SPL028) were both hybrids, and their parents were the same varieties (**Table 1**). Therefore, it was concluded that when the difference in the DNA fingerprint between two varieties is ≤1 locus, it means that the two varieties have certain similarity in species, origin, parents, flower color, etc., which is very likely to be two identical or similar varieties.

### Reliability and stability of *Lonicera* SNP fingerprints

To verify the reliability of the DNA fingerprints comprising 15 SNP loci, four samples were randomly selected from the original 39 samples, and 15 SNP loci were analyzed *via* PCR and Sanger sequencing. The 15 SNP verification results were completely consistent with the previous test results. Subsequently, genetic analysis was conducted on 10 honeysuckle samples of the same variety from different years. The results showed that the genetic stability of these honeysuckle samples was extremely strong, reaching 97.33%, a level that was consistent with the characteristics of honeysuckle as a vegetatively propagated plant. However, it is worth noting that the genotypes of the two-year-old samples “J01,” “YJ02,” and “YJ03” and the five-year-old sample “YJ07” changed from A/A to A/G in Marker 10, a result that may have been related to the second allele of the SNP. However, as a vegetatively propagated plant, the genetic traits of honeysuckle are very stable, and the probability of genetic mutation between the same varieties is very low; thus, this phenomenon was most likely caused by the duality of SNP sites. Finally, three new varieties verified the specificity of SNPs, further demonstrating the reliability of the SNPs and DNA fingerprints developed in this study.

In general, the verification results of the 15 SNP loci and the genetic stability of honeysuckle were in line with expectations; the analysis not only confirmed that the 15 SNP loci constituted the authenticity and reliability of DNA fingerprints but also verified the high genetic stability of honeysuckle, providing a data reference and theoretical support for the identification of honeysuckle germplasm and the SNP fingerprint identification method.

## Data availability statement

The original contributions presented in the study are publicly available. This data can be found here: NCBI, PRJNA895242.

## Author contributions

JL conceived of the project. JL and XC designed the experiments. XC performed most of the experiments. QH assisted in preparing seedlings for experiments. PL and XZ helped overcome technical difficulties in SNP site validation. CC, XZ, FL, and YW assisted in analyzing the data. JL, XC, and CC interpreted the data generated in the figures and wrote the manuscript. All authors contributed to the article and approved the submitted version.
