# Supplementary figures and images for: Construction of SNP fingerprint and population genetic analysis of honeysuckle germplasm resources in China
Source: Front Plant Sci. 2023 Mar 2;14:1080691. doi: 10.3389/fpls.2023.1080691 (PMC10017979; doi:10.3389/fpls.2023.1080691)

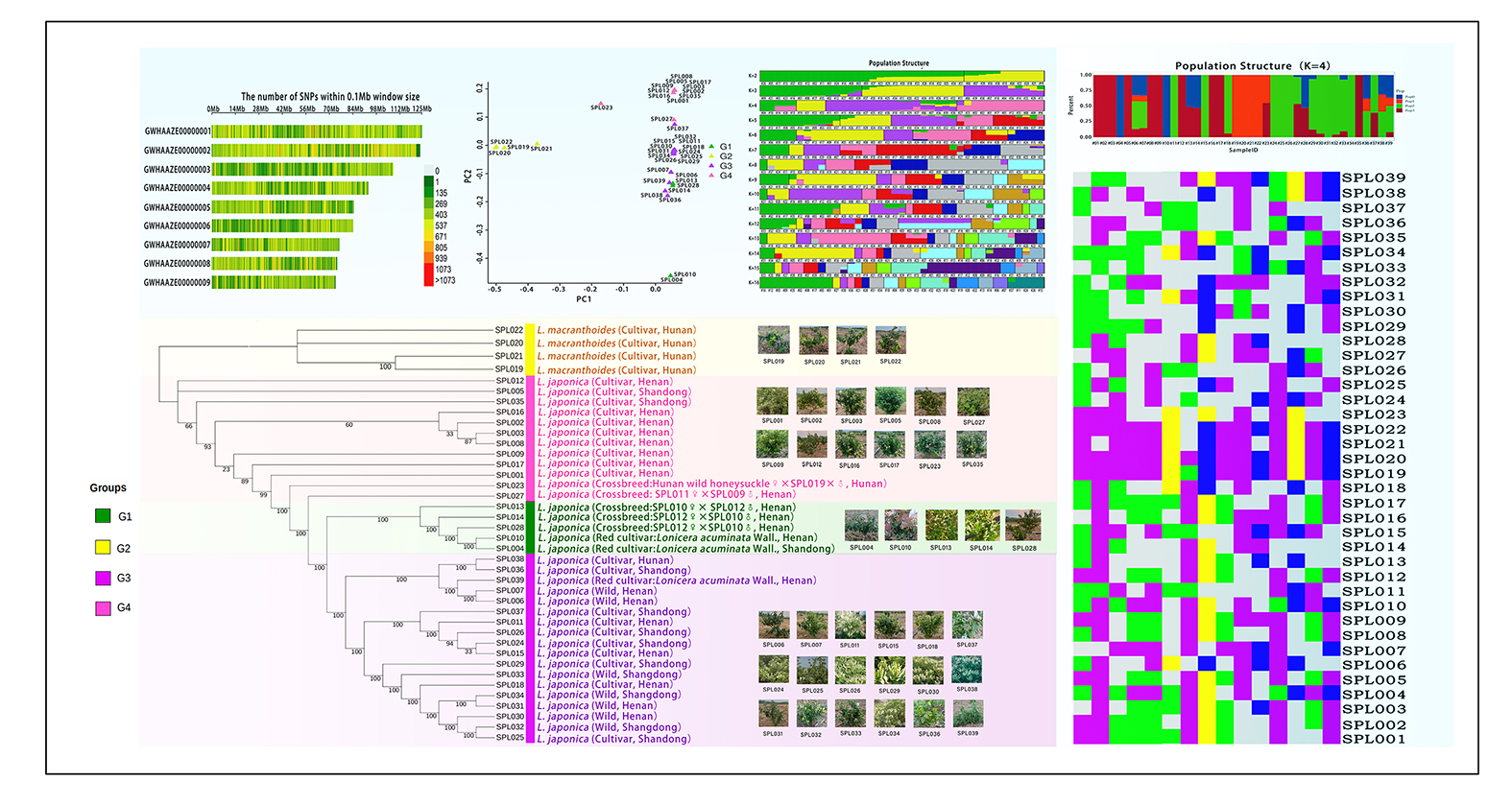

Supplement: Supplementary file 1 [file Image_1.jpeg]

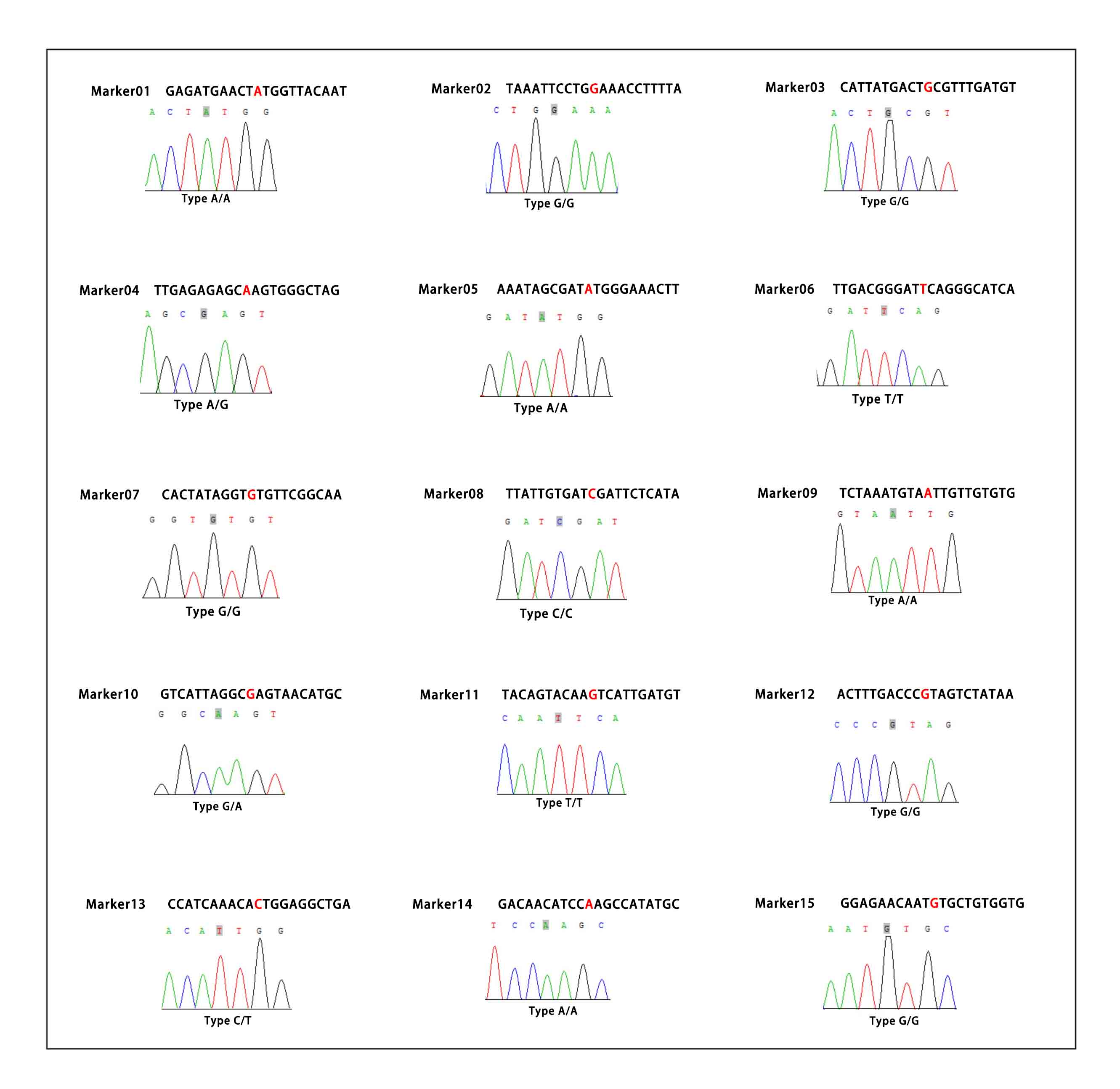

Supplement: Supplementary file 2 [file Image_2.jpeg]
